# Breast Cancer Knowledge and Screening Barriers Among Female University Students of Pakistan

**DOI:** 10.3390/ijerph23081068

**Published:** 2026-08-18

**Authors:** Sehar Iqbal, Abdul Momin Rizwan Ahmad, Zoha Imtiaz Malik, Muhammad Irfan, Taima Qudah, Michael Kundi

**Affiliations:** 1 College of Pharmacy, Al Ain University, Abu Dhabi Campus, Abu Dhabi P.O. Box 112612, United Arab Emirates; sehar.iqbal@aau.ac.ae (S.I.); taima.alqudah@aau.ac.ae (T.Q.); 2 Department of Human Nutrition and Dietetics, NUST School of Health Sciences, National University of Sciences & Technology (NUST), Sector H-12, Islamabad 44000, Pakistan; abdul.momin@nshs.nust.edu.pk (A.M.R.A.); zoha.imtiaz@nshs.nust.edu.pk (Z.I.M.); 3 Department of Health Sciences, University of York, York O10 5DD, UK; 4 Department of Statistics, Liaquat National Hospital and Medical College, Karachi 74800, Pakistan; irfanzafar892@gmail.com; 5 Department of Environmental Health, Center for Public Health, Medical University of Vienna, Kinderspitalgasse 15, 1090 Vienna, Austria

**Keywords:** breast cancer, knowledge, signs and symptoms, barriers, Pakistan

## Abstract

**Highlights:**

**Public health relevance—How does this work relate to a public health issue?**

- Breast cancer tends to increase the global burden of cancer and risk of mortality in women of all ages.
- South Asia, and especially Pakistan, faces the challenge of increasing breast cancer incidence and mortality.

**Public health significance—Why is this work of significance to public health?**

- Low screening coverage, delayed diagnosis, and gaps in provision of cancer treatment appear to be responsible for this situation in Pakistan.
- We found poor knowledge about breast cancer symptoms and risk factors among female university students, indicating even lower awareness among the general public, which could be a major obstacle for appropriate health-seeking behavior and early detection.

**Public health implications—What are the key implications or messages for practitioners, policy makers and/or researchers in public health?**

- This study will help to target educational activities, overcome knowledge barriers and increase health-seeking behavior in women.
- Socio-cultural taboos, psychological fears, and misconceptions have to be addressed in shaping breast cancer awareness programs in Pakistan.

**Abstract:**

Breast cancer is a leading public health concern among low- and middle-income countries, primarily due to inadequate knowledge and lack of early detection practices. This study aimed to assess knowledge about the signs and symptoms, risk factors, and screening procedures for breast cancer, and barriers and perceptions of screening, among female university students of Pakistan to explore the relationship between socio-demographic variables and levels of knowledge about breast cancer. A total of 413 participants took part in a cross-sectional survey and completed a self-administrative online questionnaire. The results reflected an overall poor knowledge among female students, with only 3.9% demonstrating adequate knowledge about risk factors and 7.7% correctly recognizing 80% or more relevant signs and symptoms. Embarrassment (58.8%) and lack of confidence in communicating symptoms with healthcare providers (50.6%) were the most commonly reported barriers for breast cancer screening. Young age and income status below PKR 50,000 per month (OR = 3.47, 95%CI: 1.20–10.07 rel. to highest income) were significantly associated with poor breast cancer knowledge. In conclusion, this study reports sub-optimal breast cancer awareness levels in the target population, indicating the need for awareness programs and policy-driven interventions to reduce barriers, improve knowledge, and improve adherence to screening recommendations.

## 1. Introduction

Breast cancer is one of the leading causes of female mortality. It is a major public health issue and appears to increase the global cancer burden for women of all ages. Global estimates for 2022 indicated that a total of 2.3 million new cases and 666,000 deaths can be attributed to the disease [1]. Trends in modifiable and non-modifiable risk factors, including aging, urbanization, reproductive behavior, obesity, sedentary lifestyle and dietary factors, contribute to the increase in breast cancer incidence [2]. Therefore, many studies have now emphasized the importance of preventive measures such as screening, early detection, primary management, and awareness campaigns regarding breast cancer in women [3].

The prevalence and severity of breast cancer demonstrate inequitable trends among high-income countries (HICs), and low-middle-income countries (LMICs). Incidence rates are higher in HICs, attributed to higher rates of screening and early detection, while LMICs have higher breast cancer mortality rates, mainly due to lack of adequate early diagnostics, quality treatment, and healthcare management services [4]. Recent advances in early detection, especially of female cancers [5,6], will be challenging for LMICs concerning their implementation. Furthermore, research has highlighted the importance of culturally appropriate awareness measures and education, which need to be included in LMIC health systems for cancer control and prevention [7].

South Asia, similar to other LMIC regions, faces the challenge of increasing breast cancer incidence. The health system in South Asian countries is fragile, and current issues include low screening coverage, delayed diagnosis, and gaps in provision of cancer treatment. This array of factors worsens breast cancer outcomes and prognosis in comparison to HICs. Recent GLOBOCAN analyses and other regional data have predicted that the breast cancer situation may get worse in this region due to the growing threats of population growth, and demographic and epidemiological transitions resulting in lifestyle shifts [8]. Many of these secular changes will contribute to an increase in the prevalence of breast cancer risk factors. These factors also underscore the need to design and implement cost-effective public health intervention in order to promote knowledge and awareness, reduce breast cancer stigma, and “nudge” the women towards early help-seeking behaviors [3].

Pakistan has recently been reporting an alarming number of breast cancer cases and deaths. The country’s breast cancer fact sheets placed breast cancer as the most common cancer among Pakistani women. Pakistan has also recently emerged as the country with the highest breast cancer incidence and mortality in Asia [9]. The cumulative risk of developing breast cancer in Pakistani females by the age of 75 years is reported at 11.6%. Hence, it can be estimated that 1 in 9 Pakistani females will develop breast cancer at some point in their lives. The age-standardized breast cancer incidence and mortality rate for Pakistani females are 34.2 and 18.6 per 100,000, respectively [10]. Additionally, the numbers for late presentation and diagnosis are equally alarming, with a high proportion of women being diagnosed at late stages of breast cancer. A local study reported that 70% were diagnosed at stage III or IV of breast cancer, while 4% or less were diagnosed at stage I. This pattern is consistent with low screening coverage, lack of healthcare services, socio-cultural barriers to seek diagnosis and treatment, and financial constraints [11].

Breast cancer knowledge, attitude, and self-examination practices are major determinants of early diagnosis. However, LMICs, including Pakistan, have poor levels of awareness regarding breast cancer. Knowledge of signs and symptoms and about breast self-examination (BSE) is poor. A national survey conducted to assess the prevalence of overall breast cancer awareness in young women reported poor knowledge and awareness of both the disease itself and its contributing risk factors [12]. This highlights the need for strategies to promote breast cancer awareness and knowledge among women belonging to different ages and educational backgrounds. Limited breast cancer awareness, social stigma associated with the disease, misconceptions and taboos, fear of being diagnosed, and high treatment costs all could deter females from seeking medical help even when they identify alarming initial signs and symptoms. Studies have identified female university students as a core group, to be targeted with breast cancer education initiatives, that then could transfer this information to diverse communities [13].

Increasing prevalence of breast cancer in Pakistan, which has now the highest incidence rate in Asia, coupled with indications of poor knowledge about all aspects of breast cancer among young women, build the rationale for systemically assessing the current awareness levels in female university students. Despite previous research in LMICs and South Asia indicating low breast cancer awareness levels, the main focus in these studies was knowledge about signs and symptoms and risk factors. Evidence about perceived barriers and knowledge of BSE and screening practices is lacking. Therefore, there is a need for an integrated research approach, assessing all contributing factors, including knowledge, awareness, screening barriers, and socio-demographic determinants. Such knowledge is necessary to tailor educational interventions.

## 2. Materials and Methods

### 2.1. Study Design and Target Population

For this study, a descriptive cross-sectional survey was conducted to assess knowledge, awareness and perceived barriers toward breast cancer screening among female university students in Pakistan. The study was a collaborative effort between Al Ain University, United Arab Emirates; the National University of Sciences & Technology (NUST), Pakistan; and the Medical University of Vienna, Austria. Data were collected using a structured, self-administered questionnaire developed by the research team after reviewing relevant literature on breast cancer awareness and screening practices [12]. This self-administered Google Form (Google LLC, Mountain View, CA, USA) comprised 4 parts: (i) Knowledge about breast cancer—items on signs and symptoms (taken from BC-AM [14]), risk factors (general, gynecological/obstetric, lifestyle-related), and age groups at higher risk (partly overlapping with BC-AM [14]); (ii) Awareness and barriers toward breast self-examination and screening—assessed knowledge of screening methods, frequency of self-examination, and perceived barriers to clinical breast examination and mammography; (iii) Awareness-raising strategies—explored participants’ opinions on effective methods to improve breast cancer awareness in the community; and (iv) Socio-demographic information including age, marital status, educational level, monthly family income, and family history of breast cancer. Parts (ii) and (iii) had to be designed for the specific situation in Pakistan [12]. The questionnaire was checked by a group of 5 experts from Pakistan (gynecologists, oncologists, and psychologists).

### 2.2. Eligibility Criterion for Study Participants

The target population included female students currently enrolled in undergraduate and postgraduate programs at selected universities in Pakistan. Female participants, aged 18 years or older, without any chronic disease and willing to provide informed consent, were considered eligible. Students with prior diagnosis of breast cancer were excluded. A convenience sampling strategy was used to recruit participants. The survey was developed in English and pilot-tested among a small group of students (*n* = 18) to ensure clarity and comprehension. Minor revisions were made before final distribution.

Sample size considerations were based on the following assumptions: prevalence of poor knowledge overall: 75% (based on [12]); odds ratio that should be detected: 2; prevalence of the factor under consideration: 65%; R^2^ from other variables: 0.2; (two-sided) alpha error: 5%; power: 80%. Under these conditions, the sample size must be *n* = 368. Considering a participation rate of around 60%, about 600 students were contacted by email via university email groups and student networks.

### 2.3. Data Collection and Ethical Considerations

The questionnaire was distributed online through Google Forms. Participation was voluntary, and respondents could withdraw at any stage without consequence. Informed consent was obtained electronically before accessing the survey. Confidentiality and anonymity of data were strictly maintained. Responses were collected anonymously, and no personal identifiers were stored. Ethical approval for the study was obtained from the Institutional Review Board (IRB) and ethical committee of NUMS university, Pakistan (6/IRB &EC/NUMS/34), on 21 November 2022.

### 2.4. Statistical Analysis

Data were analyzed using IBM SPSS Statistics version 27 (IBM Corp., Armonk, NY, USA). Descriptive statistics were applied to summarize participant characteristics and survey responses. Means and standard deviations (SD) were calculated for quantitative variables, while frequencies and percentages are presented for categorical variables. Knowledge scores were computed separately for signs and symptoms (11 items), risk factors (17 items), and the overall score (28 items).

The overall knowledge, attitude, and practice scores were converted into percentage scores and categorized according to Bloom’s cut-off criteria [15]. Scores of 80% and above were considered good, scores between 60% and 79% were considered moderate, and scores below 60% were considered poor. (1) Signs and Symptoms (0–11 items): Poor (0–6), Moderate (7–8), Good (9–11); (2) Risk Factors (0–17 items): Poor (0–10), Moderate (11–13), Good (14–17); and (3) Overall Knowledge (0–28 items): Poor (0–16), Moderate (17–22), Good (23–28). Furthermore, Cronbach’s alpha for the 11-item signs and symptoms sub-scale was 0.80, and 0.73 for the 17-item risk factors scale. A collective Cronbach alpha value of 0.852 was obtained for the overall score, showing an acceptable internal consistency.

Chi-square/Fisher’s exact test was applied to determine associations between qualitative variables. Binary logistic regression was performed to identify predictors of poor knowledge regarding signs and symptoms, risk factors, and overall breast cancer knowledge. Results are expressed as odds ratios (OR) with 95% confidence intervals (CI), goodness-of-fit was tested by applying the Hosmer–Lemeshow test, and a *p*-value ≤ 0.05 was considered statistically significant.

## 3. Results

- Socio-demographic characteristics of the study participants:

Characteristics of the study participants are shown in Table 1. A total of 413 participants completed the questionnaire, of which 85.5% were undergraduate students aged 18 to 22 years. Of the 413 participants, family income was below PKR 50,000 in 16.7%, 9.4% were married and 28.8% had a family history of breast cancer (Table 1).

- Knowledge about breast cancer signs, symptoms and risk factors

The mean knowledge score for signs and symptoms was 4.00 ± 2.86, with good internal consistency (Cronbach alpha = 0.803) (Table 2). A majority of participants recognized the presence of a breast lump (65.4%) and pain in the breast or armpit (63.9%) as breast cancer symptoms. However, nipple rash (16.0%), change in nipple position (15.0%), and redness (16.0%), puckering or dimpling of the skin (18.9%) were the least identified signs and symptoms.

The mean knowledge score for breast cancer risk factors was 6.51 ± 3.51, with a Cronbach alpha of 0.730, indicating acceptable internal consistency. A past history of breast cancer (59.1%), exposure to high chest radiation during childhood or adolescence (57.1%), and prolonged use of oral contraceptive pills (55.2%) were the most often identified breast cancer risk factors, while high red meat consumption (16.0%), low intake of fruits and vegetables (19.4%), and high fat/unhealthy dietary patterns (26.6%) were the least identified risk factors, as shown in Table 2.

**Figure 1:**
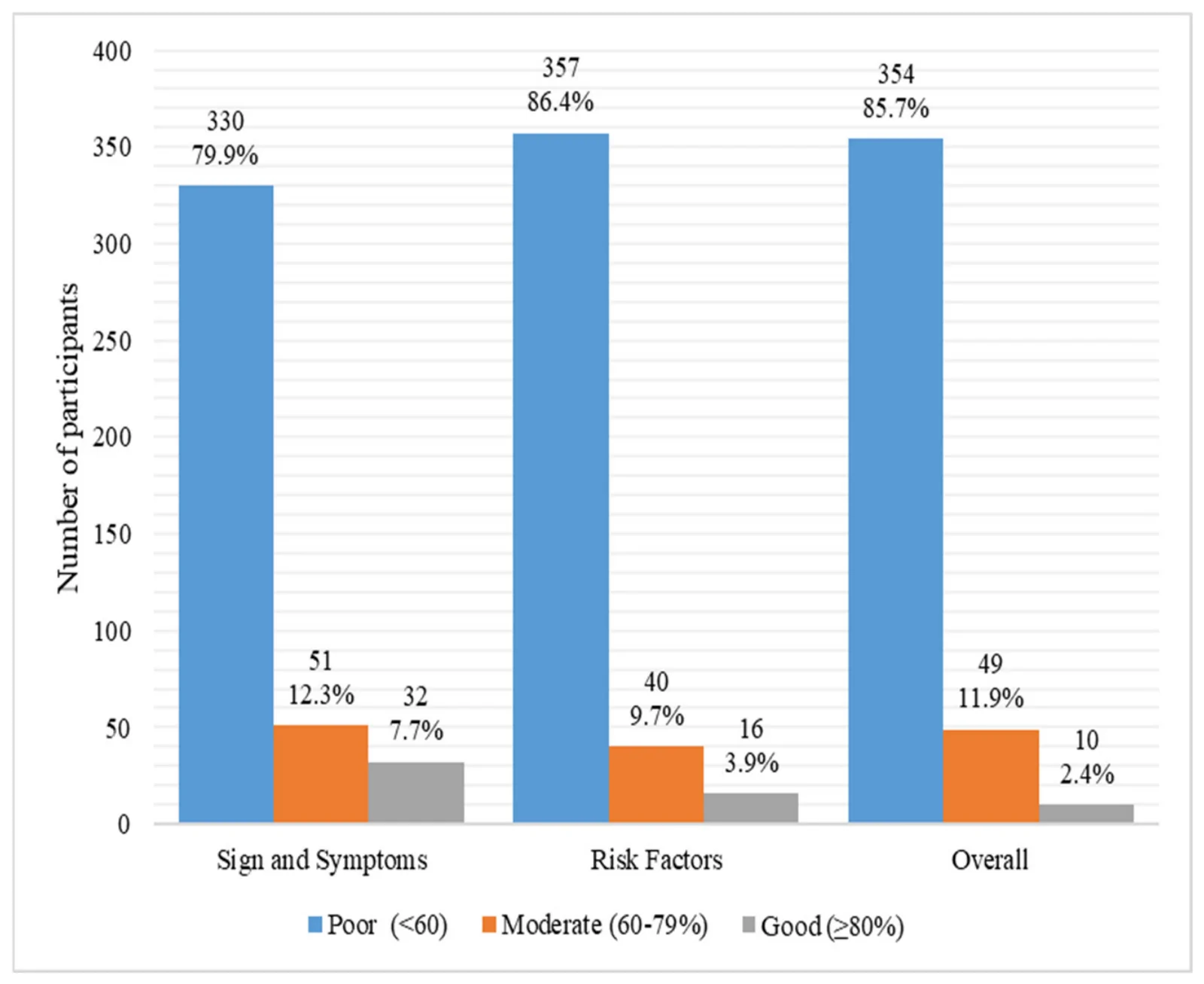
Distribution of breast cancer knowledge about signs and symptoms, risk factors, and overall knowledge (classification according to Bloom’s criteria as a percentage of knowledge items correctly identified).

Combining the two aspects, signs/symptoms and risk factors, revealed a Cronbach alpha = 0.852. Of the 413 study participants, 79.9% were categorized as having poor knowledge of breast cancer signs and symptoms, 86.4% as having poor knowledge of risk factors, and 87.5% as having overall poor knowledge of the disease (Figure 1).

- Awareness and attitudes about breast cancer screening

Regarding awareness and attitudes, less than half (45.8%) of participants were able to identify the age range at which there is an increased risk for breast cancer diagnosis (Table 3). Only 35.4% of participants reported that monthly breast self-examination (BSE) was important, while almost half (45.3%) said it should be done every 6 months. The majority (96.9%) believed that screening is useful for early detection and 77.2% of participants believed that breast cancer screening should be conducted by trained healthcare professionals. About half of the participants (50.4%) said BSE should start at age 20 and 45.3% said mammograms should be done at age 30 and above.

- Knowledge about breast cancer barriers

Many participants believed that fear of results (45.0%), embarrassment (58.8%) and lack of confidence in discussing symptoms with a doctor (50.6%) are the most important barriers regarding breast cancer screening. However, transport difficulties (9.2%) or appointment scheduling (15.0%) were less frequently reported barriers. A majority of participants identified adequate education on how to perform BSE (67.8%), community awareness programs (53.3%), and encouragement by doctors (51.3%) as the best strategies to improve breast cancer awareness. (Table 4).

We found no significant association of breast cancer knowledge with age (*p* = 0.059), marital status (*p* = 0.450), education level (*p* = 0.050), family income (*p* = 0.146) or breast cancer in family (*p* = 0.062).

Using univariate logistics regression, it was found that participants with age 18–22 years were more likely to have poor breast cancer knowledge in comparison with participants aged > 22 years (OR = 1.749, *p* = 0.062). It was found that participants who had ever been married were more likely to have poor knowledge in comparison with those who never married (OR = 1.509, *p* = 0.453). Undergraduate students (OR = 2.225, *p* = 0.025) were more likely to have poor breast cancer knowledge in comparison with students with post-graduate education or above. Participants with family income below PKR 50,000 (OR = 3.471, *p* = 0.022), PKR 50,000 to 100,000 (OR = 1.983, *p* = 0.078), PKR 100,000 to 150,000 (OR = 1.356, *p* = 0.472) and PKR 150,000 to 200,000 (OR = 1.464, *p* = 0.391) were more likely to have poor breast cancer knowledge in comparison with those having family income more than PKR 200,000. Detailed results of association and odds ratios are presented in Table 5.

## 4. Discussion

We found that most of the university students had poor knowledge of breast cancer. Yet, the presence of a breast lump and pain in the breast or armpit were the most often correctly identified features among breast cancer signs and symptoms. Similar to our findings, other studies reported a poor knowledge about the disease among university students [12,16], indicating that a knowledge gap exists across educational institutions, despite differences in academic settings and provision of health information. Studies from many other HICs and LMICs reported better knowledge regarding breast cancer among health care professionals [17,18]; this can be explained by their formal medical education and training regarding breast cancer, which is not provided to non-health professionals. Since students from disciplines other than health care can still be considered better informed about cancer, and breast cancer in particular, it can be assumed that the general public has very poor knowledge that is likely far below the level we found in female university students.

Data on breast cancer knowledge can be assessed by different study instruments. Our questionnaire is largely based on the Breast Cancer Awareness Measure (BC-AM), which collects data on breast cancer symptoms, self-examination practices, and age-related risk perception. The instrument has been used widely in public health research [14]. This tool, regarding screening practices and knowledge about screening facilities, has been developed for the situation in UK and had to be adapted to the conditions in Pakistan to provide information about attitudes to breast cancer screening and its barriers.

Regarding risk factors, a history of breast cancer in the family, exposure to radiation during childhood or adolescence, and prolonged use of oral contraceptives were the most often identified risk factors. In agreement with our findings, a study from Gaza observed that university students identified family history as an important determinant related to breast cancer and that such an encounter appears to increase breast cancer awareness [19], maybe because genetic predisposition to cancer is a widely understood concept. Conflicting findings were reported in a university-based study from Pakistan, which reported that less than 30% (29.8%) of students identified breast cancer in first-degree relatives as a risk factor [12]. Regarding contraception techniques, use of contraceptive pills has not been discussed in families or academic settings in Pakistan due to cultural constraints [13]. Socio-cultural taboos often shape health-seeking behaviors in South Asian countries, explaining the limited awareness and discussions surrounding reproductive health issues [20,21]. However, our study participants believed that the use of contraceptives might be a crucial factor related to the incidence of breast cancer. Several studies found that multiple environmental and external factors appear to increase cancer risks. Risk of breast cancer has been associated with both endogenous and exogenous estrogen levels, with hormone replacement therapy and contraceptive pills being the primary sources for exogenous estrogen [7,21], which can further be linked to the emerging breast cancer prevalence among younger women. Because estrogens are potent mitogens, exogenous estrogens can accelerate tumor growth, leading to symptoms and diagnosis at an earlier age, or even promote growth of tumors that would never have been diagnosed during the life-time of the woman.

Adequate knowledge about screening and risk factors might help to reduce disease risks and contribute to early detection, with consequences for treatment options and mortality. A majority of participants believed that screening is a useful tool for early detection and should be undertaken by professional healthcare providers. Yet, less than half of the participants were able to correctly identify the importance of age and BSE. A recent systematic review evaluating the factors related to breast cancer screening reported that family history, socio-demographic variables, knowledge, perception, self -care and social support are the most important factors for increasing the screening status around the world [22]. This evidence highlights the multifactorial nature of breast cancer screening behaviors, and solely increasing awareness is often not enough to see a noticeable difference in screening practices. Psychosocial and structural interventions across settings are needed to influence screening behavior. A study from Turkey found that fear of cancer, anxiety about mammograms, lack of a spouse or family support, and concern related to confidentiality are negatively associated with breast cancer screening propensity [23]. These findings highlight the significant impact of psychological and social factors in accessing healthcare, even in countries where easy access exists. However, emotional barriers may outweigh the accessibility and availability of healthcare.

Considering the breast screening barriers, many study participants believed that embarrassment, lack of confidence in discussing symptoms with a doctor, and fear of results were important barriers related to breast cancer screening. Other studies from Pakistan found that stigma about breast cancer, aversion of male doctors, dependence on spiritual healing, hesitancy due to lack of social acceptance, and sparsity of medical facilities are barriers regarding breast cancer screening [16]. The similarity across Pakistani studies highlights the significant role of cultural factors and gender disparities in terms of health seeking, coupled with an inadequate healthcare system that further inhibits access to healthcare services. A recent systematic review of LMICs concluded that screening costs, unavailability and distance from screening centers are the major impediments associated with low mammographic screening rates [24]. This finding aligns with the view of the students in our study, and emphasizes that even in the presence of knowledge and awareness, financial constraints, availability and psychological issues may limit access.

The “Global guidelines for breast cancer screening study” proposed that mammographic screening specifically for women aged between 50 and 69 years, and magnetic resonance imaging (MRI), should be conducted on an annual basis for women at risk of developing breast cancer [25]. Moreover, health promotion, appropriate diagnosis, and availability of social support can reduce the global burden of breast cancer [26]. The majority of participants in our study stated that education adequate to perform BSE, community awareness programs, and appropriate counseling from doctors might help to improve breast cancer awareness.

In terms of breast cancer screening practices, we found that although a majority (45.8%) correctly identified the largest risk at ≥50 years, risk at a younger age was largely underrated. These findings are consistent with a systematic review reporting only 36.3% females had awareness of diagnostic modalities of breast cancer including screening, and only 15.3% had ever undergone a breast exam [27]. Similarly, lack of knowledge regarding screening timing, starting at 20–30 years old, reflects an overall poor pattern of preventive health literacy. Additionally, psychological factors such as embarrassment (58.8%), lack of confidence communicating with clinicians (50.6%), and fear of diagnosis (40.7%) were commonly reported barriers to seeking preventive healthcare. These findings align with an exploratory study reporting that stigma, fear of social consequences, spiritual concerns, and feminine sensitivity impact health-seeking decisions [28]. Furthermore, lack of screening facilities available in public health care settings in Pakistan further acts as a barrier to breast cancer prevention and management. Currently, only 9.5% and 4.8% of urban and rural women, respectively, have access to breast cancer screening, contributing to elevated breast cancer incidence and prevalence rates [29].

Such barriers may play a role in shifting health-seeking behavior from preventive to therapeutic, prompting individuals to access health care when they observe symptoms. Overall, these results signify the role of socio-cultural taboos, psychological fears, and misconceptions in shaping breast cancer awareness and perceptions.

In addition to awareness and knowledge, we identified an association between different socio-economic variables and breast cancer awareness. Students from low-income families had poorer breast cancer knowledge. Younger participants, aged 18–22 years, were more likely to have poor knowledge about the risk factor as compared to middle-age groups. Increasing education level was associated with better knowledge. Also, students with a family history of breast cancer had a tendency for better knowledge of breast cancer. Comparable to our findings, earlier studies found that literacy status, family history and socio-economic status are significantly associated with breast cancer knowledge [30]. A Karachi-based study on undergraduate students found a high (97%) proportion of female students had heard of breast cancer, and 78% possessed good knowledge about the disease and its examination. However, only 24.9% actually performed a breast self-exam on themselves. This is consistent with comparable studies finding that, despite the high level of awareness regarding breast cancer, actual preventive practices remain low [31].

The similarity in findings suggests a simultaneous role of educational and personal experiences regarding breast cancer in shaping awareness, and perhaps also preventive and screening practices across different population groups, thereby stressing the importance of targeted interventions. Early detection, proper knowledge and educational intervention among women were shown to increase the awareness regarding breast cancer [32]. Moreover, adequate policy making in regard to availability of healthcare facilities, including screening centers and community awareness programs, might help to improve early detection and reduce mortality due to breast cancer. Future research in different geographical regions should help to identify the most underserved groups regarding breast cancer prevention.

### Study Limitations

The study has some limitations which should be acknowledged when interpreting the study’s results. Firstly, participants were recruited using convenience sampling, which may add selection bias, as those recruited may have a higher awareness of the topic under study, thereby limiting the study’s generalizability and representativeness. Furthermore, the sample was limited to university students with access to the internet, which limits the applicability of findings to the rural and low socio-economic female population. Regardless, the study provides evidence to inform breast cancer awareness initiatives in university students. However, studies involving the overall female population are needed to draw conclusions regarding breast cancer interventions for the overall Pakistani female population. Secondly, only female university students were invited and, therefore, the results do not reflect the general population. However, it can be assumed that university students, being better educated, have a better knowledge and higher awareness. Thirdly, the cross-sectional study design limits the interpretation of associations as causal, and hence, the observed relationships should be cautiously interpreted. Although our questionnaire is based on the validated BC-AM, some parts were specifically developed for the situation in Pakistan and only validated by experts. Should one be interested in the knowledge and views of the general population, our sample of young, educated women is insufficient to draw conclusions about this much larger and heterogeneous population. However, employing an a fortiori argument, our sample can be considered as the tip of the iceberg, and the huge mass of ignorance can be considered a challenging target for educational and behavioral policies. Furthermore, the knowledge levels for the study were categorized using Bloom’s criteria, a commonly used tool in KAP studies globally, but with limited psychometric validation in Pakistan. Hence, the results regarding participants’ knowledge must be interpreted with caution, as Bloom’s criteria thresholds may influence the proportion of participants in each category.

## 5. Conclusions

We found poor knowledge about breast cancer risk factors and signs and symptoms among female university students of Pakistan. Age, education and income status were associated with breast cancer knowledge.

A complex interplay of different factors, including cultural factors, lack of social support, embarrassment about discussing the issue, and fear of disease, affects screening status in Pakistan in the view of female university students. Due to Pakistan being the country with the highest breast cancer incidence and mortality rate in Asia, there is an immediate need to prioritize breast cancer awareness campaigns for the country’s population. Moreover, strategic development of adequate health facilities, easy-to-access screening centers, and low- or no-cost screening services might help improve early disease detection. In concomitance, policy making for health promotion, educational interventions and identification of barriers might help to reduce the disease burden. It is crucial to raise unsettling questions, and not to postpone comprehensive planning, with a perspicacious approach to evaluate and tackle the present and future breast cancer challenges for vulnerable societies in the country. It is high time that cost-effective healthcare strategies, comprehensive policies, and a wide range of medical infrastructural investments are planned to reduce mortality due to breast cancer.

## Figures and Tables

**Table 1:** Socio-demographic characteristics of study participants (*n* = 413).

Variables	Frequency (%)
**Age**	
18–22 years	307 (74.3)
22–26 years	89 (21.5)
26–30 years	9 (2.2)
>30 years	8 (2.0)
**Marital status**	
Ever Married	39 (9.4)
Single	374 (90.6)
**Education level**	
Undergraduate Student	353 (85.5)
Postgraduate student	45 (10.9)
PhD student	7 (1.7)
Other	8 (1.9)
**Family income**	
Below 50,000 Pak Rupee	69 (16.7)
50,000 to 100,000 Pak Rupee	133 (32.2)
100,000 to 150,000 Pak Rupee	72 (17.4)
150,000–200,000 Pak Rupee	64 (15.5)
More than 200,000 Pak Rupee	75 (18.2)
**Breast cancer in family**	
Yes	119 (28.8)
No	294 (71.2)

**Table 2:** Knowledge regarding breast cancer signs/symptoms and risk factors.

Breast Cancer Knowledge Questions	Frequency (%)
**Signs and symptoms** **(Knowledge score = 4.00 ± 2.86 ^1^, 36.4% ± 26.0% of maximum, Cronbach Alpha = 0.803)**	
Discharge or bleeding from nipple	160 (38.7)
Lump or thickening in breast	270 (65.4)
Lump or thickening under armpit	185 (44.8)
Pain in breasts or armpit	264 (63.9)
Changes in shape of breast or nipple	194 (47.0)
Changes in size of breast or nipple	164 (39.7)
Redness of breast skin	66 (16.0)
Pulling in of nipple	144 (34.9)
Change in the position of nipple	62 (15.0)
Nipple rash	66 (16.0)
Puckering or dimpling of breast skin	78 (18.9)
**Risk factors** **(Knowledge score = 6.51 ± 3.51 ^1^, 38.3% ± 20.7% of maximum, Cronbach Alpha = 0.730)**	
Past history of breast cancer	244 (59.1)
Longterm use of HRT (Hormone Replacement Therapy)	163 (39.5)
Having one or more close relatives with breast cancer	163 (39.5)
High radiation to the chest or breast in childhood or adolescence	236 (57.1)
Having used oral contraceptive pills more than 5 years	228 (55.2)
Given birth for the first time after age 30	140 (33.9)
Not having a childbirth experience	85 (20.6)
Started menstruating before age 12	88 (21.3)
Entering menopause after age 55	87 (21.1)
Low physical activity	158 (38.3)
Overweight and obesity	196 (47.5)
Not having breastfed	139 (33.7)
Smoking or alcohol consumption in the past or present	179 (43.3)
Stress and anxiety	151 (36.6)
High consumption of red meat	66 (16.0)
Low consumption of vegetables and fruits	80 (19.4)
High consumption of fatty foods/Unhealthy eating	110 (26.6)
**Overall** **(Knowledge score = 10.51 ± 5.84 ^1^, 37.5% ± 20.9% of maximum, Cronbach Alpha = 0.852)**	

^1^ mean ± SD, range for signs and symptoms: 0–11, for risk factors: 0–17, for overall: 0–28.

**Table 3:** Awareness and attitudes regarding breast cancer and screening methods.

Question	Answer	Frequency (%)
As much as you know, at which age women are most likely to have breast cancer	Less than 30-year-old woman	145 (35.1)
	at 50 years or above	189 (45.8)
	at 70 years or above	1 (0.2)
	Do not know	78 (18.9)
In your opinion, breast self-examination should be done…	Rarely or never	25 (6.1)
	At least once every 6 months	187 (45.3)
	At least once a month	146 (35.4)
	At least once a week	55 (13.3)
* Clinical breast cancer screening should be done by …	trained health professional every year	319 (77.2)
	mammogram every 2 years	141 (34.1)
	mammogram or breast examination by trained health professionals	42 (10.2)
Breast cancer screening is a useful tool for early detection of breast cancer	Surely yes	294 (71.2)
	Likely yes	106 (25.7)
	Likely no	8 (1.9)
	Surely no	5 (1.2)
The best time to first go to breast examination for breast cancer is	At 20 years	208 (50.4)
	At 30 years	143 (34.6)
	At 40 years	47 (11.4)
	At 50 years	13 (3.1)
	At 60 years or later	2 (0.5)
Best time to start mammography is	At 20 years	121 (29.3)
	At 30 years	187 (45.3)
	At 40 years	80 (19.4)
	At 50 years	22 (5.3)
	At 60 years or later	3 (0.7)
During the menstrual cycle, the best time to do a self-breast exam is	One week after the onset of menstruation	164 (39.7)
	Two weeks after the onset of menstruation	102 (24.7)
	Three weeks after the onset of menstruation	40 (9.7)
	Shortly before onset of menstruation	63 (15.3)
	During menstruation	44 (10.7)

* Multi-select item.

**Table 4:** Barriers and perception to increase awareness of breast cancer (multi-select items).

Barriers for Breast Cancer Screening	Frequency (%)
Worrying about what the doctor might find	186 (45)
Too many other things to do	86 (20.8)
Too scared	168 (40.7)
Too embarrassed	243 (58.8)
Too busy to find time to go to the doctor	137 (33.2)
Difficulty to make an appointment with doctor	62 (15)
Not feeling confident talking about my symptom with the doctor	209 (50.6)
Difficulty to arrange transport to the clinic	38 (9.2)
Having difficulties to talk to the doctor	152 (36.8)
Worried about wasting the doctor’s time	37 (9)
**Methods to increase awareness and understanding of the importance of breast screening**	
Encourage participation in breast self-examination by the doctor	212 (51.3)
Each woman how to perform breast self-examination	280 (67.8)
Have free screening available at basic health units	196 (47.5)
Hold classes or lectures on breast cancer	196 (47.5)
Establish awareness programs in the community	220 (53.3)
Conduct screening programs in shopping malls and shopping areas	95 (23)
Making screening compulsory when women are coming for regular checkups	159 (38.5)

**Table 5:** Association between breast cancer knowledge and socio-demographics of participants.

Predictors	Poor Knowledge About Breast Cancer			
	Frequency (%)	*p*-Value ^1^	OR (95% CI)	*p*-Value ^2^
**Age**				
18–22 years	269 (87.6%)	0.059	1.749 (0.973–3.143)	0.062
22–26 years	85 (80.2%)		Ref	
**Marital status**				
Ever Married	35 (89.7%)	0.450	1.509 (0.516–4.413)	0.453
Single	319 (85.3%)		Ref	
**Education level**				
Other	8 (100.0%)	0.050	NA	NA
Undergraduate	307 (87.0%)		2.225 (1.105–4.480)	0.025
Postgraduate or above	39 (75.0%)		Ref	
**Family income**				
Below 50.000 Pak Rupee	64 (92.8%)	0.146	3.471 (1.197–10.066)	0.022
50.000 to 100.000 Pak Rupee	117 (88.0%)		1.983 (0.927–4.242)	0.078
100.000 to 150.000 Pak Rupee	60 (83.3%)		1.356 (0.591–3.110)	0.472
150.000–200.000 Pak Rupee	54 (84.4%)		1.464 (0.612–3.503)	0.391
More than 200.000 Pak Rupee	59 (78.7%)		Ref	
**Breast cancer in family**				
Yes	96 (80.7%)	0.062	0.582 (0.328–1.033)	0.065
No	258 (87.8%)		Ref	

OR, odds ratio; CI, confidence interval; NA, not available; Ref, reference category; ^1^ Chi-square/Fisher exact test; ^2^ Wald test from binary logistic regression.

## Data Availability

All data supporting reported results can be found in the article. Original data may be obtained by reasonable request from the corresponding author.

## Abbreviations

The following abbreviations are used in this manuscript:

BC-AM	Breast Cancer Awareness Measure
BSE	Breast Self-Examination
GLOBOCAN	Global Cancer Observatory
HICs	High-Income Countries
LMICs	Low- and Middle-Income Countries
HRT	Hormone Replacement Therapy
OR	Odds Ratio
CI	Confidence Interval
IRB	Institutional Review Board
